# Venetoclax-Based Combinations in Acute Myeloid Leukemia: Current Evidence and Future Directions

**DOI:** 10.3389/fonc.2020.562558

**Published:** 2020-11-05

**Authors:** Bachar Samra, Marina Konopleva, Alessandro Isidori, Naval Daver, Courtney DiNardo

**Affiliations:** ^1^ Department of Leukemia, The University of Texas MD Anderson Cancer Center, Houston, TX, United States; ^2^ Hematology and Stem Cell Transplant Center, AORMN Hospital, Pesaro, Italy

**Keywords:** venetoclax, acute myeloid leukemia, venetoclax-based combinations, relapsed acute leukemia, acute myeloid leukemia combination therapy

## Abstract

The past decade has witnessed major advances in our understanding of molecular biology, which led to breakthrough novel therapies, importantly including the B-cell lymphoma-2 (BCL-2) inhibitor venetoclax. Notably, venetoclax-based combinations have improved outcomes, including both remission rates and overall survival, of older patients with acute myeloid leukemia (AML) deemed “unfit” for intensive chemotherapy due to age or comorbidities. This has translated into a rapid and widespread use of venetoclax-based combinations in both academic and community-based settings. Other venetoclax-based combinations are being investigated in AML with the ultimate goal of improving cure rates across many subgroups; frontline and relapsed/refractory, in combination with intensive chemotherapy, in the post-transplant setting, or as maintenance strategy. In this article, we summarize the current available data on venetoclax-based combinations. We also highlight areas of unmet medical need, and we offer practical clinical pearls for management of patients receiving such therapy.

## Introduction

Acute myeloid leukemia (AML) is a disease of older patients; the incidence increases with age and the median age at diagnosis is 68 years (1). AML is a very heterogeneous disease characterized by many chromosomal translocations and genetic mutations due to the abnormal proliferation and differentiation of a clonal population of myeloid stem cells (2, 3). Standard therapy for fit and primarily younger patients (age < 65 years) consists of intensive induction chemotherapy (anthracycline combined with cytarabine) to achieve complete remission (CR) followed by consolidative high-dose cytarabine regimens or hematological stem cell transplant (HSCT) (4). Despite high remission rates; ~80% in younger patients and ~50% in older patients, most patients eventually relapse and succumb to their disease. Even among younger fit patients with favorable disease biology, cure rates do not exceed 60–70% (excluding acute promyelocytic leukemia) (5). This has been attributed mainly to the persistence of residual chemo-resistant leukemic cells, termed “measurable residual disease” (MRD), referring to a low-level of disease that is below the detection threshold of conventional cyto-morphological assessment (6). Outcomes are especially poor in older patients, which account for the majority of AML cases, with only 5–10% long term survival, primarily due to patient-related factors that may preclude the use of intensive chemotherapy or myeloablative HSCT, and/or disease-related factors that are associated with resistance to therapy (**Table 1**) (3). Historically, there has not been an optimal treatment approach for older patients with AML that are poor candidates for intensive induction chemotherapy. These patients will be referred to as “unfit” in this review. The mainstay of therapy in this population has been “lower-intensity” therapies including low-dose cytarabine (LDAC) and hypomethylating agents (HMA); azacitidine (AZA) and decitabine (7–9). Despite the survival benefit of these therapies compared with supportive care alone, outcomes are dismal with rates of CR or CR with incomplete count recovery (CRi) of 20–30% and median overall survival (OS) rates <12 months. However, the past decade has witnessed major advances in our understanding of the disease biology and the mutational landscape, allowing for the development of novel therapies that have improved outcomes. Since 2017, eight new drugs have been approved by the U.S. Food and Drug Administration (FDA) for the treatment of AML, including the *FLT3* inhibitors midostaurin and gilteritinib, the *IDH* inhibitors ivosidenib and enasidenib, the anti-CD33 monoclonal antibody gemtuzumab ozogamicin, liposomal daunorubicin and cytarabine (CPX-351), the hedgehog pathway inhibitor glasdegib and the B-cell lymphoma-2 (BCL-2) inhibitor venetoclax (10–19). The combination of venetoclax with either HMA or LDAC has received accelerated FDA approval trials for newly diagnosed (ND) patients with AML older than 75 years or unfit for intensive chemotherapy, based on two multicenter independent early phase clinical trials. This advance is considered by most experts to be the most impactful of all other new approvals for such population with high unmet need, with favorable safety profile and dramatic improvement in CR, MRD negativity and OS rates, compared with historical controls (20). This has translated into fast and widespread incorporation of venetoclax-based therapies both in academic and community settings. In this comprehensive review, we focus on the role of venetoclax-based combination therapies in AML, including the current evidence and future directions. Importantly, while the AML community gains more experience with venetoclax-based therapies, the level of comfort among many physicians in managing such regimens remains relatively limited. We provide here practical considerations including dose modifications, drug‐drug interactions, treatment duration, and antimicrobial prophylaxis that may be safely applied in a real-world setting.

**Table 1:** Challenges in treating AML in older patients.

Clinical factors
Decreased performance status
Increased number of comorbidities
Decreased organ function
Polypharmacy
Frequent dose reductions, delays, or omission
Higher risk of adverse events (infections, neurotoxicity, secondary malignancies)
Antecedent hematologic disease (myelodysplastic syndrome, myeloproliferative neoplasm)
Prior exposure to chemotherapy or radiation
**Cytogenetic/molecular factors**
Increased incidence of adverse-risk karyotype (e.g., -5, -7, 3q26 aberrations, t(6;9), 11q23 aberrations except for t(9;11), “monosomal karyotype”
Lower incidence of favorable-risk karyotype (e.g. core-binding factor)
Increased incidence of adverse-risk molecular signatures (*TP53*, *FLT3*-ITD, *MLL* rearrangement)
**Social factors**
Inadequate caregiver and/or social support
Transportation/travel difficulties to tertiary centers
**Other factors**
Perceived lack of benefit of receiving anti-leukemia therapy rather than supportive care

## Mechanism of Action and Preclinical Data

**Figure 1:**
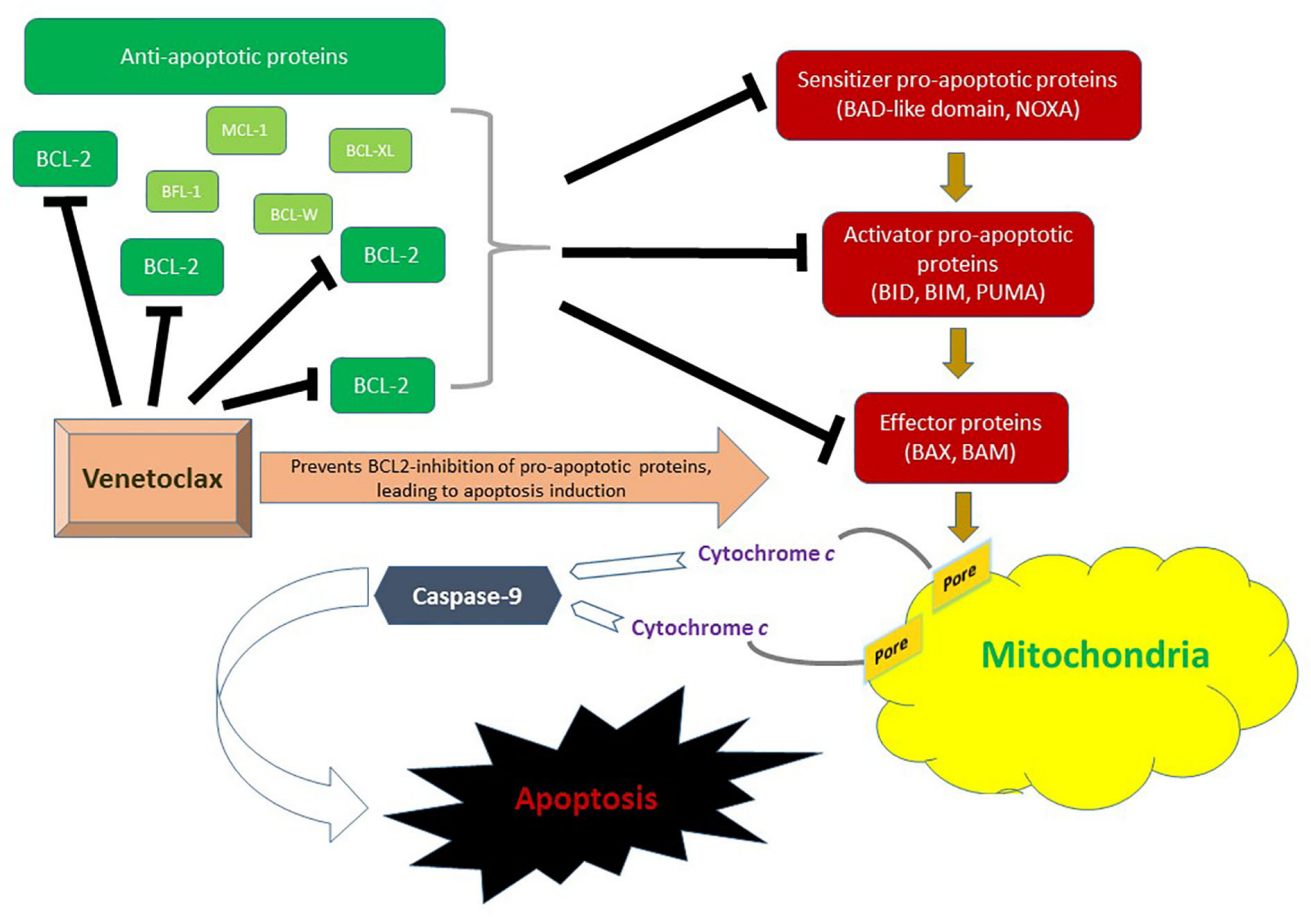
Role of the BCL-2 family in the mitochondrial (intrinsic) pathway of apoptosis. BAX and BAM are the principal effectors of the intrinsic apoptotic pathway. Their activation through pro-apoptotic activator (BID, BIM, and PUMA) and sensitizer (NOXA) proteins leads to permeabilization of the mitochondrial outer membrane. This results in the release of cytochrome *c* into the cytosol thus triggering activation of apoptosis-inducing caspase cascade *via* caspase-9. Anti-apoptotic proteins include BCL-2, MCL-1, BCL-XL, BCL-W and BFL-1. In AML, BCL-2 is upregulated. Venetoclax inhibits BLC-2 and therefore prevents BCL-2 mediated inhibition of pro-apoptotic pathway molecules BAX and BAK, ultimately promoting cell death.

The BCL-2 family consists of 18 different pro-apoptotic and anti-apoptotic molecules that are key regulators of the intrinsic (mitochondrial) apoptotic pathway and have been implicated in the tumorigenesis and cell survival of many hematological malignancies (21). There are three functional groups; anti-apoptotic proteins (BCL-2, MCL-1, BCL-XL, BCL-W, BFL-1), pro-apoptotic BCL-2 homology domains 3 (BH3) [BIM, BID, BAD, PUMA, NOXA, BIK, BMF, HRK], and effector proteins (BAX, BAK). In response to stress or DNA damage, the intrinsic pathway is activated, leading through BAX and BAK effector proteins to formation of pores in the outer mitochondrial membrane. This results in the release of cytochrome *C* into the cytosol, thus activating caspase-9, and ultimately triggering proteolytic cell death. **Figure 1** summarizes the role of BCL-2 family in the mitochondrial apoptotic pathway.

The overexpression of BCL-2 in hematologic malignancies has been associated not only with enhanced cell survival and apoptosis evasion, but also with therapy resistance, especially in leukemic stem cells (6). Navitoclax is the first in-class oral BCL-2 (and BCL-XL) dual inhibitor that showed antileukemic activity in chronic lymphocytic leukemia (CLL), however, its further development has been limited by its target specific (BCL-XL) dose-limiting severe thrombocytopenia (22). Venetoclax is an oral BH3 mimetic highly selective for BCL-2 without targeting BCL-XL, with dramatic activity in CLL, notably independent of *TP53* mutation (23–25). Early pre-clinical studies have shown that AML cells, especially leukemic stem cells, are dependent on BCL-2 for survival, and inhibition by venetoclax can lead to rapid apoptosis of AML cells and eradication of quiescent leukemic stem cells (26–29). Moreover, synergistic antileukemic activity with HMA and chemotherapy, which have apoptotic function as well, has been demonstrated in preclinical models providing rationale for clinical combination strategies (30, 31).

## Single-Agent Activity in AML

The safety and efficacy of single‐agent venetoclax in AML was first evaluated in a phase 2 study of 32 patients with high-risk relapsed/refractory (R/R) disease, or AML unfit for intensive chemotherapy (32). The median age was 72 years (range 19–84). The CR/CRi rates were 19%, and an additional 19% of patients experienced a partial bone marrow response. Rates of response were higher among patients with *IDH* mutations (36%). Venetoclax monotherapy showed an acceptable safety profile with neutropenic fever being the most common grade 3/4 adverse event (AE). There was no tumor lysis syndrome (TLS) or treatment-discontinuation due to therapy. BH3 profiling confirmed on-target BCL-2 inhibition. The safety of single agent venetoclax with (albeit modest) antileukemic activity prompted further studies of combining venetoclax with other active agents demonstrating synergy in pre-clinical models.

Data on the single agent activity of venetoclax in the frontline setting is more limited. The CAVEAT trial, which combined venetoclax for 14 days with standard induction with cytarabine + idarubicin showed that treatment-naive *NPM1-* and *IDH2*-mutant blasts are highly sensitive to venetoclax monotherapy (33). After a 7-day venetoclax monotherapy pre-phase, 30% of patients had bone marrow blast reduction of >50%, mainly representing cases of *NPM1* (median of 56%) and *IDH2* mutations (56%), and less so in *TP53* (17%) and *FLT3-*ITD (7%) mutations.

## Venetoclax-Based Combinations

### Hypomethylating Agents (HMA)

Preclinical studies have shown synergistic induction of cell death between HMA and BCL-2 inhibitors. Azacitidine may decrease MCL-1 level which can mediate resistance to BCL-2 inhibitors. Through NOXA induction, azacytidine may also “prime” AML cells for venetoclax-induced apoptosis. Additionally, azacitidine and BCL-2 inhibitor synergistically activate BAX and mitochondrial apoptosis in AML cells (30, 31). Accordingly, venetoclax was combined with HMA therapy in a pivotal phase Ib clinical trial of 145 untreated patients >65 years and unfit for chemotherapy. Factors for unfitness for chemotherapy included age >75 years, limited performance status and/or organ dysfunction (cardiac, pulmonary, renal, or hepatic disease). Patients who received prior HMA therapy and those who had favorable-risk disease cytogenetics were excluded. The median age was 74 years (range 65–86). Half of patients had poor-risk cytogenetics and a quarter had secondary AML (s-AML). Patients received venetoclax 400, 800, or 1,200 mg daily in combination with either decitabine or azacitidine at standard dosing. The 400 mg dose was chosen for expansion. Combination therapy was well tolerated, with most common side effects being gastrointestinal symptoms, fatigue, and neutropenia. Main common grade 3–4 AEs were infections (45%). Notably, the 30-day induction mortality was low at 3% and no TLS was observed. The CR/CRi rates were 67% and did not differ between azacitidine and decitabine. The median time to response was 1.2 cycles (months) and the MRD negativity rate among responders was 29%. With a median follow up of 15 months, the median duration of response (DOR) and OS were 13.1 and 17.5 months, respectively. These results compare favorably with historical cohorts treated with HMA monotherapy where CR/CRi rates are 30%, median time to response is 3.5 cycles, and median OS is <12 months. Among patients in CR/CRi, median DOR was 11.3 months and median OS was not reached (NR). Although benefit was seen in all patients, outcomes differed between molecular and cytogenetic subgroups. Accordingly, CR/CRi rates were higher in patients with *NPM1* and *IDH1/2* mutations (91 and 71%, respectively) and lower in patients with *TP53* mutations and poor cytogenetics (47 and 60%, respectively). Median DOR was also longer in patients with *NPM1* and *IDH1/2* mutations (NR for both), and shorter for patients with *FLT3* and *TP53* mutations (11 and 5.6 months, respectively). Survival was especially favorable for patients with *NPM1* and *IDH1/2* mutations (median OS NR and 24.4 months, respectively) and especially poor for patients with *TP53* mutation (median OS 7.2 months).

The confirmatory international phase 3 placebo-controlled randomized trial (VIALE-A; NCT02993523) of azacitidine +/- venetoclax in a similar patient population was just presented at the European Hematology association (EHA) 2020 virtual meeting with positive results and then published in the New England New Journal of Medicine (34). Among 431 patients treated (286 with AZA + venetoclax, and 145 with azacytidine), the median age was 76 years; 25% had s-AML. The trial met its primary endpoint of improved OS with the combination. With a median follow-up of 20 months, the median OS was improved from 9.6 months (7.4–12.7 months) with azacitidine to 14.7 months (11.9–18.7 months); HR 0.66 (95% CI 0.52–0.85), P < 0.001. The CR/CRi rate and median DOR were improved from 28.3 to 66.4% (P < 0.001) and 13.9 months to 17.8 months with azacytidine and venetoclax-combination, respectively. Such results support the combination of venetoclax + azacytidine as the new standard of care for older unfit patients with newly diagnosed AML and are expected to grant venetoclax full FDA approval in this setting.

In order to expand upon the therapy and indications for HMA + venetoclax combinations, venetoclax is currently being evaluated in combination with 10-day decitabine in a phase 2 trial at MD Anderson Cancer Center (MDACC) for patients older than 60 years and ineligible for intensive chemotherapy in the ND as well as R/R settings (NCT03404193). Prior HMA therapy [for myelodysplastic syndrome (MDS)] is allowed. Decitabine is given for 10 days in induction and for 5 days per cycle after remission. Venetoclax is administered daily until marrow remission, then decreased to 21 or 14 days per cycle in subsequent cycles to allow for augmented hematological recovery. Use of *FLT3* inhibitors is allowed in patients with *FLT3* mutations. Early results on 101 patients have been presented at the American Society of Hematology (ASH) 2019 annual meeting (35). The median age was 70 (range 34–75) and 2/3 of patients had adverse-risk disease. More prolonged myelosuppression was identified with this regimen leading to the recommendation to hold VEN on day 21 in the setting of a leukemia-free marrow, nevertheless induction mortality was low (2.5% in ND disease, and 5% in R/R disease). This underscores the importance of mitigation strategies to manage venetoclax-related cytopenia. The CR/CRi rates were different according to disease type; 95% in ND AML, 67% in s-AML, 37% in previously treated s-AML (with HMA for MDS), and 27% in R/R AML. The MRD negativity rate among responders was notably high (70%). Twenty percent of patients were able to undergo hematopoietic stem cell transplant (HSCT), with no mortality observed at day-100 post-HSCT. With a median follow up of 8 months, the 6-month OS was 90% in ND AML, 56% in s-AML, 62% in treated s-AML, and 53% in R/R AML. Similar to the prior study, outcomes differed by molecular subgroups confirming superior efficacy in patients with *NPM1, IDH1/2* as well as a signal for improved response in *RUNX1* mutations. Interestingly, despite less CR/CRi rates in patients with R/R disease and *NPM1* mutations (60% compared with 100% in ND setting), median OS was NR in both cohorts. Outcomes of patients with *TP53* mutation will be discussed below (*TP53* section). An update with additional patients and longer follow-up is anticipated later this year.

### Low-Dose Cytarabine (LDAC)

Venetoclax was also combined with LDAC in another pivotal phase Ib/II trial of patients with ND AML >60 years and ineligible for intensive chemotherapy (19). Prior HMA therapy for antecedent MDS was allowed. LDAC was given at a standard dose of 20 mg/m2 once daily for 10 days and the venetoclax recommended phase 2 dose was 600 mg daily. Among 82 treated patients, 32% had poor-risk cytogenetics and 50% had s-AML, 60% of which had prior HMA. The median age was 74 years (range 63–90). Combination therapy was well tolerated with the most common grade 3 AEs being cytopenias and infections. The CR/CRi rate was 54%, with a median time to response of 1.4 cycles, and the MRD negativity rate was 32%. Higher responses were seen in *de novo* AML; CR/CRi rate of 71% and median DOR of 11.6 months; versus 35% and 8.1 months, respectively in s-AML. Patients with *NPM1* or *IDH1/2* mutations again had better outcomes, with CR/CRi rates of 89 and 72%, respectively, compared to 30 and 44% for patients with *TP53* or *FLT3* mutations. The median OS was 10.1 months with an estimated 1-year OS of 27%. Overall, these results appear similar to the experience of venetoclax with HMA especially when excluding patients with prior HMA.

More recently, the confirmatory VIALE-C phase III randomized trial, of LDAC with or without venetoclax has been published (36). Eligibility criteria were similar to the phase Ib/II trial. In total, 211 patients were randomized in a 2:1 ratio to either venetoclax (n = 143) or placebo (n = 68). Median age was 76 years (range 36–93), and 38% had s-AML, half of them had prior HMA. A third of patients had poor-risk cytogenetics and *TP53* and *FLT3* mutations were present in 20 and 18%, respectively in the venetoclax arm. The addition of venetoclax to LDAC resulted in a 25% survival benefit arm [hazard ratio (HR) 0.75, P = 0.11], which was not statistically significant; with a median OS of 7.2 months and 4.1 months, respectively. Although unexpected, the lack of statistical significance for venetoclax survival benefit could be explained, at least partly, by two main reasons. First, the expectations for the survival benefit may have been overestimated as the study was designed to detect a 45.5% reduction in mortality with 90% power and a significance level with two-sided alpha of 0.05. Second, the authors reported a high number of administratively censored patients on the venetoclax arm (12 versus 6% within the first 6 months) as enrollment was still ongoing as early as 3.4 months before the pre-planned OS analysis. Accordingly, an unplanned analysis with an additional 6 months of follow up, with the majority of patients now censored beyond the median OS time, demonstrated statistical significance for OS with a median of 8.4 months for the venetoclax arm (HR 0.70; P = 0.04). In multivariable analysis, venetoclax was an independent predictor for better OS (HR 0.67, P = 0.03) as well as age <75 years, better performance status, *de novo* AML (versus s-AML), and intermediate cytogenetics (versus poor-risk cytogenetics). Other clinically meaningful endpoints also favored venetoclax-combination. The CR/CRi rates were 48 and 13% for the venetoclax combination and LDAC alone, respectively (P < 0.001). Additionally, event-free survival and transfusion independence both favored venetoclax arm (4.7 months versus 2.0 months, P = 0.002, and 37 versus 16%, P = 0.002, respectively). Although bone marrow MRD assessment at the time of CR was not mandated, the MRD negativity rate with venetoclax among responders was notably low (6%). There were no new safety signals in this study. Main grade 3/4 AEs in the venetoclax arm were febrile neutropenia (32%), neutropenia (47%), and thrombocytopenia (45%). Induction mortality was fairly high; 13% with venetoclax + LDAC and 16% with LDAC, possibly explained by the relatively high-risk study population, as 60% were older than 75 years of age and 50% had ECOG performance status 2–3.

### Intensive Chemotherapy

Pre-clinical data have shown synergy between cytotoxic agents, including cytarabine, and venetoclax by enhancing BH3 activity and/or suppressing MCL-1 to promote apoptosis. Sequestration of inducers of apoptosis (Bim) has been suggested as mechanism of resistance to BCL-2 inhibitors. The combination with cytotoxic agents may overcome such resistance by upregulating Bim (37). Ongoing early phase trials have evaluated the combination of venetoclax with intensive chemotherapy for fit patient with AML (**Table 2**). A phase I trial of escalating dose of venetoclax in combination with standard 7 + 3 induction (7-day cytarabine and 3-day anthracycline) has shown that 200 mg daily for 4 days can be safely given in patients with ND AML 18–60 years of age (44). Dose escalation to 400 mg is ongoing and plans are underway to compare 7 + 3 with or without venetoclax in a randomized phase 3 trial. The phase Ib CAVEAT trial is combining venetoclax with 5 + 2 induction chemotherapy (5-day cytarabine and 2-day anthracycline) for older (age > 65 years) fit patients with ND AML (39). Of the 44 patients treated thus far, 41% had s-AML and 38% had prior HMA. The median age was 72 years (range 63–80). Venetoclax was evaluated in dose escalation cohorts of 50–600 mg daily for 14 days with a 7-day dose ramp-up followed by chemotherapy. Consolidation is given for 4 cycles consisting of 14-day venetoclax combined with 2-day cytarabine and 1-day anthracycline chemotherapy. Venetoclax is given as maintenance in 14-day cycles every 28 days for 7 cycles. The CR/CRi rate was 71% overall; 95% in patients with ND AML and 42% in those with s-AML. Patients with adverse cytogenetics or prior exposure to HMA had lower response rates (46 and 43%, respectively). As observed in prior studies, response rates were over 90% in patients with *NPM1*, *RUNX1*, or *IDH* mutations but only 33% in patients with *TP53* mutation. Induction mortality was 7%, and no TLS was observed. These interim results confirm the safety of the combination of venetoclax up to 600 mg, with reduced-dose induction chemotherapy for fit elderly patients with AML.

**Table 2:** Summary of published prospective studies on venetoclax-based combinations in AML.

Combination	Phase	Population	N	Median age [range], years	30-day mortality, %	CR/CRi rate, %	MRD negativity, %	Median DOR, months	Median OS, months	Reference
HMA	Ib	ND Age >75 years or unfit for chemotherapy	145	74 [65–86]	3	67 *De novo*: 67 s-AML: 67	29	11.3 *De novo*: 9.4 s-AML: NR	17.5 *De novo*: 12.5 s-AML: NR	(18)
Decitabine (10-day in induction then 5-day after CR/CRi)	II	ND or R/R Age >60 unfit for chemotherapy	101	70 [34–75]	ND: 2.5 R/R: 5	*De novo*: 95 s-AML: 67 ts-AML: 37 R/R: 27	70	*De novo*: 8.5 s-AML: 6.3 ts-AML: 4.8 R/R: 6.6	6-month rate: *De novo*: 90% s-AML: 56% ts-AML: 62% R/R: 53%	(34)
HMA	Ib	ND Age >18 years post HMA failure	22	76 [41–92]	NA	41	NA	NA	5.5 12.5 if CR	(38)
LDAC	Ib/II	ND Age >60 years unfit for chemotherapy	82	74 [63–90]	6	54 No HMA: 62 Prior HMA: 33	NA	8.1	10.1 No HMA: 13.5 Prior HMA: 4.1	(19)
LDAC vs LDAC alone	III	ND Age >75 years or unfit for chemotherapy	142	76 [36–93]	13 vs. 16	48 vs. 13	6 vs 1	NA	8.4 versus 4.1 (P = 0.04)	(36)
5 + 2	Ib	ND Age >65 years and fit for chemotherapy	44	72 [63–80]	7	71 *De novo*: 95 s-AML 42	NA	NA	*NPM1*: 12.5 *IDH2*: NR *IDH1*: 6 *TP53*: 4 *FLT3*-ITD: 6	(33, 39)
FLAG-IDA	Ib/II	ND or R/R Adults fit for chemotherapy	40 ND: 14 R/R: 26	47 [21–72]	ND 0 R/R 13 (60-day)::	ND: 85 R/R: 72	ND: 85 R/R: 50	NA	ND: NR R/R: 10	(40)
Cladribine + LDAC, alternating with Azacitidine	II	ND Age >60 years or unfit for intensive chemotherapy	35	69 [57–84]	0	89	84		65% (1-year)	(41)
Idasanutlin	Ib	R/R Age >60 years	71	72 [60–93]	6	29	45	5	6	(42)
Cobimetinib	Ib	R/R Age >60 years	71	72 [60–93]	NA	18	NA	NA	NA	(43)

AML, acute myeloid leukemia; HMA, hypomethylating agent; ND, newly diagnosed; R/R, relapsed/refractory; LDAC, low-dose cytarabine; 5 + 2, 5-day cytarabine + 2-day anthracycline; FLAG-IDA, fludarabine, cytarabine, growth-stimulating factor, idarubicin; CR, complete remission; CRi, complete remission with incomplete count recovery; NA, not available; NR, not reached; s-AML, secondary acute myeloid leukemia; ts-AML, treated secondary acute myeloid leukemia.

The second study is a single institution phase Ib/II combining venetoclax with FLAG-IDA (fludarabine, cytarabine, granulocyte colony–stimulating factor [G-CSF] and idarubicin) for adult patients with ND or R/R AML that are fit for chemotherapy (NCT03214562). Initially, venetoclax was given for 21 days. However, due to high responses but increased AEs including neutropenic fever and sepsis, the protocol was amended to reduce the duration of venetoclax to 14 days and reduce the dose of cytarabine from 2 to 1.5 g/m^2^. Improved safety was seen with this dosing regimen leading to the recommended phase 2 induction dosing is as follows: Venetoclax 400 mg daily on days 1–14, G-CSF 5 mcg/kg daily from days 1–7 (or one dose of PEG-filgrastim after day 5 to replace the remaining doses, fludarabine 30 mg/m^2^ daily from days 2–6, cytarabine 1.5 g/m^2^ daily from days 2–6 and idarubicin from days 4–6 (8 mg/m^2^ daily in ND disease and 8 mg/m^2^ daily in R/R disease). Bone marrow assessment is done at the end of cycle 1, between day 21 and 28. Consolidation may be given up to 6 cycles, with only 3 days of fludarabine and cytarabine and typically with only one additional consolidation cycle including more anthracycline. Interim results have been presented at ASH 2019 annual meeting (40). Forty-four patients have been treated thus far with a median age of 47 years (range 21–72). Among patients with ND AML (14 patients), no early death was seen and the CR/CRi and MRD negativity rates were both 85%. Median time to best response was 27 days (range 20–40). Among patients with R/R AML (26 patients), the 60-day mortality was 13%. The CR/CRi and MRD negativity rates were 72 and 50%, respectively. Among patients with *TP53* mutation (n = 5; 4 with R/R AML and 1 with ND AML), the CR/CRi rate was 60%. With a median follow-up of 5.5 months, median OS was NR in patients with ND AML and 9.4 months in patients with R/R AML. Of interest, all 5 patients with MLL rearrangement responded (3 R/R AML and 2 ND AML), and underwent subsequent HSCT; 4 of them are alive after a median of 8 months.

A phase II trial is currently investigating the addition of venetoclax to a lower-intensity backbone regimen consisting of 2 cycles of cladribine with LDAC alternating with 2 cycles of azacitidine in older (age > 60 years) or unfit patients with ND AML. Venetoclax is given for 21 days in induction and 14 days after remission (NCT03586609). Consolidation/maintenance is given for up to 18 cycles. Thirty-five patients have been treated thus far (41). The median age was 69 years (range 57–84). The CR/CRi rate and MRD negativity rates were 89 and 84%, respectively. Responses were lower among adverse-risk karyotype (55%), which accounted for a third of patients. Interestingly, both patients with mutant *TP53* had CR. The combination was well tolerated with no early deaths and the median time to count recovery was 32 days. Follow-up remains short and the 1-year OS was 65%.

In summary, the addition of venetoclax to intensive or lower-intensity chemotherapy has shown manageable safety and encouraging efficacy. The low induction mortality in above trials is notable (0–7%) and may not be generalizable to “real world” setting as these studies are all single institution studies on a highly selected patient population with small sample size. Longer follow-up and larger randomized trials are needed to assess the relative contribution of venetoclax to induction backbones and whether such high and deep responses will translate into better survival.

### *IDH1/2* Inhibitors

*IDH1*/2 mutations occur in 15–25% of AML (45, 46). Pre-clinical and clinical studies have shown that *IDH1*/2 mutations induce BCL-2 dependence, making them particularly sensitive to venetoclax, both as single agent and in combination with other agents (18, 19, 36, 47, 48). Patients with *IDH1/2*-mutant AML treated with venetoclax-based combinations have enjoyed not only higher and more durable responses, but also longer survival compared to the global population of each study (median OS was 19.4 months and 24.4 months, with LDAC and HMA, respectively) which is on par with azacitidine + ivosidenib or azacitidine + enasidenib studies (49, 50). On the other hand, ivosidenib (*IDH1* inhibitor) and enasidenib (*IDH2* inhibitor) as monotherapies have yielded ~40% response rates and median OS of ~12 months in patients with AML harboring these mutations (12, 13, 51, 52) (**Table 3**). The high efficacy, favorable safety profiles and minimal drug-drug interactions between *IDH* inhibitors and venetoclax in *IDH1/2*-mutant AML, have generated interest in combining them together to test synergy and improve outcomes. This concept is currently being investigated in a phase Ib/II clinical trial of a combination of venetoclax and ivosidenib, with or without azacitidine in patients with *IDH1-*mutated AML (NCT03471260). Preliminary results have been presented at the American Society of Clinical Oncology (ASCO 2020 meeting for the first 18 evaluable patients treated (3 cohorts with 6 patients each, ivosidenib + venetoclax 400 mg or 800 mg +/- azacytidine), showing good tolerability and promising efficacy (53). No new safety signal was observed, and the CR/CRi rate was 89% (100% with ivosidenib + venetoclax 800 mg, 67% with ivosidenib + venetoclax 400 mg, and 67% with ivosidenib + venetoclax 400 mg + azacytidine). After a median follow up of 3.5 months, median OS was not reached in treatment naïve patients, and 9.7 months in R/R patients. This study continues to enroll to better define duration and biomarkers of response.

**Table 3:** Data on targeted-therapy trials in *IDH1/2*-mutant ND AML.

	Ivosidenib	Enasidenib	HMA + venetoclax	LDAC + venetoclax (HMA naïve)	AZA + Ivosidenib	AZA + Enasidenib	Venetoclax + ivosidenib
**N**	34	39	35	18	23	68	12
**CR/CRi rate, %**	42	30.8	71	72	70	68	83
**Time to CR (median), months**	2.8	3.7	2.1 (whole study)	1.4 (time to first response)	3.5	5	NA
**DOR (median), months**	NR	NR	NR	NR	NR	NR	NA
**OS (median), months**	12.6	11.3	24.4	19.4	NA	22.0 (NR in patients with CR)	NR in ND and 9.7 months in R/R
**Reference**	(51)	(52)	(18)	(19, 36),	(49)	(50)	(53)

ND, newly diagnosed; AML, acute myeloid leukemia; N, number of patients; CR, complete remission; CRi, complete remission with incomplete count recovery; DOR, duration of response; OS, overall survival; HMA, hypomethylating agent; LDAC, low dose cytarabine; AZA, azacytidine; NR, not reached; NA, not available.

### *FLT3* Inhibitors

Pre-clinical data have suggested that *FLT3‐*ITD mutation in AML may produce intrinsic/primary resistance to venetoclax by enhancing the expression of anti-apoptotic BCL-2 relatives such as BCL‐XL and MCL‐1 (**Figure 1**) (54, 55). Responses to venetoclax-based combinations in *FLT3*-mutant AML have not only been lower than other subgroups but have also been short-lived (18, 19, 36). The combination of venetoclax and quizartinib, a small molecular oral *FLT3*-tyrosine kinase inhibitor (TKI), induced tumor regression in *FLT3*-mutant AML cell lines that was more durable than with either agent alone, preventing tumor re‐emergence for up to 3 months following cessation of therapy (54). Additionally, venetoclax-induced apoptosis may help overcome resistance to FLT3-TKIs. Based on this synergy and priming activity, an ongoing phase Ib multi-institutional clinical trial is investigating the combination of gilteritinib, an oral *FLT3*-TKI, with venetoclax for patients with R/R AML (NCT03625505). Early results have been presented at ASH 2019 (56). Among 23 patients treated, 18 patients had mutant-*FLT3* (16 ITD and 2 TKD). The median age was 58 years (range 23–81). Median number of prior therapies was 2. Sixty percent of patients had received prior *FLT3*-TKIs and 50% had received HSCT. The combination was well tolerated, and no TLS or treatment-related discontinuation were observed. Among patients with *FLT3* wild-type, there was one early death (due to disease progression) and the CR/CRi rate was 20%. Among patients with *FLT3*-mutant AML, there were no early deaths and the CR/CRi rate was impressive at 88%. This compares favorably to the response rates seen with venetoclax and gilteritinib as single agents in a similar setting (20 and 34%, respectively). The trial continues to enroll patients in an expansion cohort using venetoclax at 400 mg daily and gilteritinib at 120 mg daily. A similar trial is investigating the combination of venetoclax with quizartinib (NCT03735875). Two independent phase I/II clinical trials at MDACC are investigating the safety and efficacy of the triplet combination of HMA + venetoclax + *FLT3*-TKIs for patients with *FLT3*-mutant R/R or ND AML unfit for chemotherapy (NCT03661307, NCT04140487).

### *TP53* Mutation

Preclinical data have shown that *TP53* deficient/mutant cells have decreased BAX protein expression, increased apoptotic threshold, and resistance to BH3 mimetics (57). The combination of BCL-2 and MCL-1 is highly synergistic and may be effective in compensating for the *TP53* defect. Patients with *TP53*-mutant AML treated with combination of venetoclax with HMA had low response rates (< 50%), and short duration of responses and poor survival (~6 months) (18, 19, 37). Increasing the duration of decitabine to 10 days in induction does not appear to overcome resistance of *TP53* mutation to venetoclax despite higher response rates at 85% (median OS was 5.8 months in ND and 4.5 months in R/R AML). Therefore, further novel approaches are needed. APR-246 is a novel agent that can induce selective apoptosis in p53-mutant cancer cells by restoring p53 conformation and activity. The combination of APR-246 with azacitidine has shown early promising activity in *TP53*-mutant MDS and AML with CR/CRi rates of 75–87%, and is being tested in an international phase 3 randomized placebo-controlled trial (NCT03745716) (58, 59). Whether the addition of venetoclax to APR-246 and azacitidine will further improve outcomes is uncertain and is being evaluated in a multicenter phase I clinical trial (NCT04214860). **Table 4** summarizes available data on novel combinations for *TP53*-mutant ND AML.

**Table 4:** Data on targeted-therapy trials in *TP53-*mutant ND AML.

	HMA + Venetoclax	LDAC + Venetoclax	AZA + APR-246	AZA + Magrolimab (anti-CD47)
**N**	35	10	11	22
**CR/CRi rate, %**	47	30	55	55
**Time to CR (median), months**	2.1 (whole study)	1.4 (time to first response)	NA	1.9
**DOR (median), months**	5.6	NA	NR	NR
**OS (median), months**	7.2	3.7	NR	NR
**Reference**	(18)	(19)	(58)	(60)

ND, newly diagnosed; AML, acute myeloid leukemia; N, number of patients; CR, complete remission; CRi, complete remission with incomplete count recovery; DOR, duration of response; OS, overall survival; HMA, hypomethylating agent; LDAC, low dose cytarabine; AZA, azacytidine; NR, not reached; NA, not available.

## Mechanisms of Resistance

Despite impressive response rates and improved survival with venetoclax-combinations, a third of patients seem to be refractory. Furthermore, outcomes remain suboptimal in patients who are R/R, post-HMA therapy, and/or in the presence of *TP53* and signaling mutations. Sensitivity to venetoclax is directly tied to the amount of BCL-2 actively binding and sequestering pro-apoptotic proteins, also known as priming, rather than BCL-2 expression (61). The most recognized mechanism of resistance to venetoclax is the upregulation of BCL-2 family anti-apoptotic proteins such as BCL-XL and MCL-1 leading to leukemic cell survival, especially in the presence of *FLT3‐ITD* or *PTPN11* mutations (32, 55, 62, 63). The inhibition of BCL-XL (with navitoclax) has been associated with severe thrombocytopenia which limited its further clinical development in AML. Based on preclinical data showing synergy between selective MCL-1 inhibitors and venetoclax, this combination may be a potential strategy to overcome venetoclax-resistance in AML and is currently being tested in clinical trials (**Table 5**) (64–67). Moreover, venetoclax has demonstrated preclinical synergistic activity with other agents that could indirectly inhibit MCL-1. These agents include the following: Cobimetinib, a mitogen-activated protein kinase (MEK) inhibitor, which downregulates MCL-1 by suppressing the MAPK signaling pathway, idasanutlin, an MDM2 inhibitor, which promotes MCL-1 degradation by activating p53, and dinaciclib or alvocidib, which are cyclin-dependent kinase 9 (CDK9) inhibitors that can lead to transcriptional downregulation of MCL-1. Clinical trials of these drugs in combination with venetoclax are ongoing (**Table 5**).

**Table 5:** Ongoing combination trials of venetoclax in AML.

Treatment	Phase	NCT	Setting
**HMA**			
10-day decitabine	II	NCT03404193	ND or R/R
**Chemotherapy**			
Cladribine + LDAC alternating with azacitidine	II	NCT03586609	ND
FLAG-IDA	II	NCT03214562	ND or R/R
7 + 3	Ib	NCT03709758	ND
Gemtuzumab ozogamicin (anti-CD33)	Ib	NCT 04070768	R/R
CPX-351	I	NCT03826992	R/R
CPX-351 lower-intensity	Ib	NCT04038437	ND
**Targeted agents**			
IMGN632 (anti-CD123)	Ib/II	NCT04086264	R/R
Cobimetinib (MEK inhibitor) or idasanutlin (MDM2 inhibitor)	I/II	NCT02670044	R/R
Lintuzumab-Ac225 (anti-CD33)	I/II	NCT03867682	R/R
Quizartinib (FLT3 inhibitor)	Ib/II	NCT03735875	R/R
Gilteritinib (FLT3 inhibitor)	I	NCT03625505	R/R
Dinaciclib (CDK9 inhibitor)	Ib	NCT03484520	R/R
Alvocidib (CDK9 inhibitor)	Ib	NCT03441555	R/R
AMG-176 (MCL1 inhibitor)	Ib	NCT03797261	R/R
S64315 (MCL1 inhibitor)	I	NCT03672695	R/R
HDM201 (MDM2 inhibitor)	I	NCT03940352	R/R
Selinexor (XPO1 inhibitor)	I	NCT03955783	R/R
Ruxolitinib (JAK2 inhibitor)	I	NCT03874052	R/R
**HMA + targeted agents**			
IMGN632 (anti-CD123)	Ib/II	NCT04086264	R/R
Gilteritinib (FLT3 inhibitor)	I/II	NCT04140487	ND or R/R
Quizartinib (FLT3 inhibitor)	I/II	NCT03661307	ND or R/R
Gemtuzumab ozogamicin (anti-CD33)	I/II	NCT03390296	ND
Avelumab (PD-L1 inhibitor)	I/II	NCT03390296	ND
Ivosidenib	I/II	NCT03471260	ND
Pevonedistat (NEDD8-activating enzyme)	I/II	NCT03862157	ND

AML, acute myeloid leukemia; HMA, hypomethylating agent; ND, newly diagnosed; R/R, relapsed/refractory; LDAC, low-dose cytarabine; FLAG-IDA, fludarabine, cytarabine, growth-stimulating factor, idarubicin; 7 + 3, 7-day cytarabine + 3-day anthracycline.

## Management During Induction

### Dosing and Drug Interactions

Based on phase I/II trials, the established venetoclax dose is 400 mg daily with HMA or chemotherapy and 600 mg daily with LDAC as higher doses have been associated with more hematological toxicity without added clinical benefit. Since venetoclax is a CYP3A4 substrate, dose modifications are needed when using other drugs metabolized by CYP3A4, including “azole” antifungals. Based on pharmacokinetic studies, the dose of venetoclax should be reduced by 50% for moderate inhibitors CYP3A4 (fluconazole, isavuconazole, ciprofloxacin, erythromycin, calcium-channel blockers) and by at least 75% for strong inhibitors (posaconazole, voriconazole, clarithromycin) (68, 69). As more pharmacokinetic data are emerging, and until we are able to measure venetoclax concentration levels in clinic, we modify the venetoclax-dose with azole as used in the VIALE-A and VIALE-C trials: 200 mg with fluconazole, 50 or 70 mg with Posaconazole, and 100 mg with other strong CYP3A4 inhibitors including voriconazole. While the original HMA + VEN clinical trial and the subsequent FDA approved schedule is daily dosing, given the high efficacy and the associated significant myelosuppression, we have found that holding venetoclax once marrow remission or aplasia is confirmed around day 21–25 has been an effective strategy to mitigate prolonged cytopenias (see response assessment below) (70).

### Prevention of TLS

TLS is a significant risk associated with venetoclax due to the profound and often rapid apoptotic effect caused by BCL-2 inhibition. Interestingly, TLS has been less frequently seen in AML than in CLL (0 and 2% in the HMA and LDAC pivotal combination studies, respectively), with low rates likely due in part to a decreased burden of disease in AML compared to CLL, as well as aggressive mitigation and monitoring strategies mandated in the AML trials. TLS risk is increased in patients with bulky disease, leukocytosis, high lactate dehydrogenase level, hyperuricemia, and underlying kidney disease. Given the higher sensitivity to venetoclax, *NPM1* and *IDH1*/2 mutations might also be additional risk factors for TLS and more caution is needed in patients harboring these mutations. Therefore, appropriate hydration and uric acid-lowering drugs (allopurinol and/or rasburicase) are indicated prior to treatment initiation. Cytoreduction with hydroxyurea to lower white blood cell count to at least 25,000 or below is also recommended. Venetoclax should be started at a lower dose and escalated in a daily ramp-up schedule according to tolerance and TLS labs. In contrast to CLL where weekly ramp-up is recommended, a 3–5-day ramp-up is reasonable and appropriate in AML. Accordingly, if the patient is not on “azole” or other CYP3 active medications, then venetoclax ramp up would be as follows: 100 mg on day 1, 200 mg on day 2, and 400 mg on day 3 (if combined with LDAC, then up to 600 mg on day 4). A dose-modified ramp-up is recommended if the patient is concurrently on CYP3A4 inhibitors as previously discussed (e.g. 50 -100-200 mg with moderate inhibitors, and 20-50-100 mg with strong inhibitors). TLS labs are recommended prior to, 8 hours after each new dose of venetoclax, and 24 h after final dose. With these aggressive mitigation strategies and depending on the pre-defined risk of individual patients, it is not unreasonable to initiate venetoclax-based therapy as outpatient, at least for lower-risk patients, according to institutional guidelines and the level of comfort of the treating physicians/staff.

### Response Assessment

Venetoclax-based combinations have yielded high, deep, and rapid CR/CRi rates especially in the frontline setting. The median time to response and best response are generally ~1 cycle and 2 cycles across trials, respectively (18, 19, 35, 36, 40). Due to the associated significant myelosuppression, an end of cycle bone marrow assessment is crucial not only to assess disease status, but also to guide duration of venetoclax therapy, dose modifications and future cycles. Therefore, a bone marrow aspirate/biopsy should be performed around 21–28 day from the start of therapy. If morphologic remission is achieved, then we recommend holding venetoclax and delaying next cycle until count recovery (absolute neutrophil count >0.5 x 10^9^/l or preferably 1 x 10^9^/l, and platelet count >25 x 10^9^/l or preferably 50 x 10^9^/l). Up to 14-day delay is appropriate and the use of G-CSF support may be warranted (see below). If there is persistent disease, then venetoclax should be continued and a second cycle should commence as scheduled, as the persisting cytopenias are more related to the active disease rather than treatment-related toxicity. The use of G-CSF is contraindicated if there is persistent disease and is discouraged within 7 days prior to the bone marrow assessment to avoid interpretation bias. If no morphologic remission is obtained after 2 cycles, then considerations for salvage options and clinical trial referral need to be initiated as late responses are less frequent.

## Management Post-Remission

### Management of Cytopenia and the Use of G-CSF

Once in remission, a routine bone marrow assessment should be done every 2–3 cycles in the first 6 months, then less frequently thereafter (e.g. every 4 months) or when clinically indicated (signs of relapse such circulating blasts or recurring cytopenia that are unexplained or out of proportion to treatment-related toxicity). Myelosuppression is very common with prolonged use of venetoclax-based combinations and nearly all patients will need dose/duration modification of venetoclax and/or the backbone therapy (e.g. HMA) at some point. In our practice, venetoclax duration is shortened by 7 days (to 14 days from 21 days, or to 21 days from 28 days) if more than 1 week was needed for count recovery or in the setting of infectious complications in prior cycle. If prolonged myelosuppression or bone marrow hypocellularity reoccurs, then the duration of venetoclax is further decreased (after ruling out relapsed disease) but to no less than 7 days per cycle. We feel especially comfortable shortening venetoclax duration in the absence of MRD in patients with durable ongoing remissions. In many cases, the use of G-CSF support (for 2–3 days) may help accelerate neutrophil recovery and avoid treatment delay or dose modification. Moreover, the backbone therapy (e.g. HMA) may be dose-reduced or shortened such as reducing azacitidine to 5 days (from 7) or to 50 mg/m^2^ (no less than 25 mg/m^2^) or decitabine to 3 or 4 days (from 5) or to 15 mg/m^2^ (no less than 10 mg/m^2^) per cycle. This is especially useful in the setting of ongoing bone marrow hypocellularity. The venetoclax-based combination therapy is given indefinitely, unless HSCT is performed, and we do not recommend stopping either venetoclax or the backbone HMA/LDAC therapy as the efficacy of monotherapy in this setting is unknown. This is being investigated in a clinical trial of younger patients with AML (NCT03573024).

### Antimicrobial Prophylaxis

Antimicrobial prophylaxis is widely used for patients with AML as infectious complications, which are strongly associated with the depth and duration of neutropenia, are a major cause of morbidity and mortality. Due to its on-target apoptotic effect, venetoclax-related neutropenia is not only nearly universal, but can also be severe, especially during induction (neutrophils < 100 x 10^0^/l). Despite optimal dose modifications and treatment delays, the median duration of neutropenia with venetoclax can range from 7 days to 25 days (71). The incidence of invasive fungal infections with venetoclax-based therapies in prospective and retrospective studies using non-azole antifungal prophylaxis (e.g. echinocandin) or no prophylaxis has ranged between 8 and 24% (18, 72). Predictors for infections included treatment in the R/R setting and lack of therapy response. ASCO in partnership with the Infectious Diseases Society of America (IDSA) have updated their guidelines in 2018 for antimicrobial prophylaxis for patients with cancer-related immunosuppression (73). A mold-active agent triazole as well as antibiotic prophylaxis with fluoroquinolone are recommended in most, if not all, patients with AML/MDS. The use of posaconazole has been shown to be superior to non-mold active agents (fluconazole or itraconazole) in terms of infectious complications and mortality in the setting of intensive induction chemotherapy (74). Although the level of evidence for the choice of antifungal prophylaxis is not as robust in the setting of venetoclax-based lower-intensity regimens, in our practice, we prioritize the use of an antiviral, fluoroquinolone antibacterial, and mold-active antifungal prophylaxis in the setting of profound (< 0.5 x 10^9^/l) and prolonged (> 7 days) neutropenia. Patients with history of severe infection or neutropenic fever, with ongoing comorbidities such as hepatic or renal dysfunction are also considered high-risk and are offered antimicrobial prophylaxis. We use echinocandin if there are contraindications for azoles (e.g. liver impairment). As previously discussed (section 6A; prevention of TLS), the dose of venetoclax should be adjusted when using azoles and ciprofloxacin.

## Hematopoietic Stem Cell Transplant

HSCT remains the most important curative modality in AML. In the pivotal phase Ib/II trials of venetoclax-based combinations with HMA or LDAC, 10% of the 304 treated patients were able to undergo allogeneic HSCT, highlighting that a sizeable minority of patients who may be deemed “unfit” at diagnosis, may later improve after response to an effective and tolerable therapy and qualify for a more intensive modality. The median age was 69 years (range 63–76). *TP53* or *FLT3* mutations were present in 40% of cases and 70% of patients were in CR/CRi prior to HSCT. Nearly 40% of transplanted patients had a remission longer than 2 years. These findings suggest that venetoclax-based induction regimens may provide a path to cure even among patients that at diagnosis, are deemed “unfit” for induction chemotherapy.

Post-HSCT AML relapse remains a major therapeutic challenge with high unmet need. Data on safety and efficacy of venetoclax-based combinations as salvage therapy after HSCT is scarce. In a retrospective study of 18 patients who had post-HSCT AML relapse (the majority within 6 months), venetoclax combined with HMA or LDAC yielded a composite CR/CRi/morphologic leukemia-free state rate of 53%. The rate of infectious complications was 73% and the median OS since venetoclax-based therapy was 130 days. Despite the challenge in distinguishing transplant related and disease- or treatment-related morbidity and mortality, the meaningful responses observed are promising and warrant further investigation in the post-HSCT setting.

## Maintenance

Venetoclax-combinations are also being evaluated as maintenance strategies either after chemotherapy or after HSCT. A phase 3 clinical trial is currently investigating the safety and efficacy of maintenance azacitidine plus venetoclax combination compared with supportive care in adult patients with AML post-HSCT (“VIALE-M”, NCT04161885). The same combination is being studied in a phase 2 clinical trial for patients with higher-risk AML in CR after chemotherapy and not immediately candidates for HSCT (NCT04062266).

## Conclusions

The advent of venetoclax-based combinations has been revolutionary in the treatment of adult patients with AML and are now considered standard of care for older patients ineligible for intensive chemotherapy. However, therapy does not appear to be curative, at least for the majority of patients; many new questions have emerged and others remain unanswered. Current and future studies are awaited to determine whether venetoclax-based lower-intensity regimen can replace intensive chemotherapy induction. Many subgroups continue to have suboptimal outcomes including patients with R/R disease, therapy-related AML, prior HMA exposure or post-HSCT relapse. Mechanisms of clonal resistance are an active area of research and may provide a rationale for potential therapeutic strategies (*TP53* mutation or upregulation of MCL-1). Improvement and better standardization of the concept of MRD may further improve our treatment strategies including the possibility of an MRD-directed time limited approach. Although another major benefit for venetoclax is its oral formulation and ease of use, in the era of increasing use of expensive novel agents frequently associated with high out-of-pocket expense, and the inflation of cancer care, awareness on potential financial burden is essential, and studies evaluating cost-effectiveness of venetoclax-based combinations as well as strategies aiming to minimize financial toxicity are warranted.

## Author Contributions

BS and CD designed the manuscript. BS drafted and illustrated the manuscript. CD, MK, ND, and AI critically reviewed, revised, and approved the final manuscript. All authors contributed to the article and approved the submitted version.

## Conflict of Interest

MK received the following: consulting/honorarium from AbbVie, Genentech, F. Hoffman La-Roche, Stemline Therapeutics, Amgen, Forty-Seven, Kisoji; research funding/clinical trials support: AbbVie, Genentech, F. Hoffman La-Roche, Eli Lilly, Cellectis, Calithera, Ablynx, Stemline Therapeutics, Agios, Ascentage, Astra Zeneca, Forty-Seven; stock options/royalties: Reata Pharmaceutical. ND received research funds from Abbvie, Genentech, Astellas, Daiichi-Sankyo, BMS, Novimmune, Immunogen, Servier, Forty-Seven, Pfizer; consultancy/honoraria from Abbvie, Genentech, Jazz, Daiichi-Sankyo, Astellas, Immunogen, Trillium, Forty-Seven, Gilead, Novartis, Pfizer, Agios, and BMS. CD had and advisory role for AbbVie, Agios, Celgene, Daiichi Sankyo, ImmuneOnc, and Notable Labs.

The remaining authors declare that the research was conducted in the absence of any commercial or financial relationships that could be construed as a potential conflict of interest.

The reviewer GM declared a past co-authorship with several of the authors MK, ND and CD to the handling editor.
